# Clinician perspectives on infusion site‐of‐care shifts in pediatric inflammatory bowel disease: An anonymous national web‐based survey

**DOI:** 10.1002/jpr3.70238

**Published:** 2026-09-01

**Authors:** Brad Pasternak

**Affiliations:** ^1^ Department of Pediatric Gastroenterology Phoenix Children's Hospital Phoenix Arizona USA

**Keywords:** brown bagging, care coordination, payer mandates, specialty pharmacy, white bagging

## Abstract

Commercial payers and pharmacy benefit managers use site‐of‐care and specialty drug distribution policies (including white bagging and brown bagging) that can redirect infusion‐based biologic therapies from hospital outpatient departments to alternate settings. We conducted an anonymous web‐based survey distributed via a national pediatric gastroenterology (GI) listserv (February 13–27, 2026) to characterize perceived access barriers and decision drivers for acceptable infusion sites among pediatric inflammatory bowel disease (IBD) clinicians. Among 100 respondents, prior authorization/payer friction was most frequently rated as a major/severe pain point (66%). The highest‐rated decision drivers were reaction management capability (88% very/extremely important), reliable communication back to the GI team (88%), patient experience (88%), and pediatric infusion expertise (87%). These findings highlight administrative burden as the dominant barrier and underscore the importance of safety capability and bidirectional care integration when pediatric patients with IBD are shifted across sites of care.

## Abstract (highlights)

What Is Known

- Payers use site‐of‐care and specialty drug distribution policies to shift infusion‐based therapies away from hospital outpatient departments.
- Pediatric inflammatory bowel disease biologic infusions require pediatric‐trained staff, reaction management capability, and reliable coordination with specialty teams.

What Is New

- In a national web‐based convenience sample of pediatric gastroenterology (GI) clinicians (*N* = 100), prior authorization/payer friction was the most common major/severe site‐of‐care barrier (66%).
- Clinicians prioritized reaction management capability, bidirectional communication with the GI team, patient experience, and pediatric infusion expertise as determinants of an acceptable infusion site (approximately 87%–88% very/extremely important).

## 1 INTRODUCTION

Commercial payers and pharmacy benefit managers use site‐of‐care and specialty drug distribution policies that can redirect infused therapies from hospital outpatient departments to alternate sites of care.^1^, ^2^, ^3^ White bagging refers to the distribution of patient‐specific medication from a pharmacy (typically a specialty pharmacy) to a clinician's office, hospital, or clinic for administration, whereas brown bagging refers to dispensing directly to the patient who transports the medication to the site of administration.^4^ For pediatric patients with inflammatory bowel disease (IBD), biologic infusions often occur in pediatric hospital infusion units where staff expertise, emergency readiness, and specialty‐team communication are integrated into care.^5^ Professional guidance emphasizes that when pediatric patients receive non‐hospital biologic infusions, minimum quality standards should include pediatric‐specific training, reaction management protocols, and reliable bidirectional communication.^5^ Despite growing policy‐driven site‐of‐care shifts, national data describing pediatric gastroenterology (GI) clinicians' perceived access barriers and determinants of acceptable infusion sites remain limited.

The objective of this study was to characterize clinician perspectives on (1) current infusion site‐of‐care patterns, (2) perceived pain points as patients are shifted to non‐hospital settings, and (3) decision drivers for referral to an acceptable pediatric‐focused infusion option.

## 2 METHODS

### 2.1 Ethics statement

Phoenix Children's Hospital Institutional Review Board reviewed this project and determined it to be exempt. The survey collected no patient‐level data or protected health information. The first page of the web‐based survey included an information statement; respondents indicated consent by proceeding to the questionnaire (implied consent).

### 2.2 Survey design and administration

We developed a brief, anonymous, web‐based survey to assess pediatric GI clinicians' perspectives on infusion site of care in the setting of payer‐directed redirection from hospital‐based pediatric infusion units. Survey domains were informed by published discussions of payer site‐of‐care policies and pediatric IBD non‐hospital infusion quality safeguards.^1^, ^2^, ^3^, ^4^, ^5^ The instrument included respondent characteristics, current infusion site‐of‐care patterns, perceived access barriers (pain points), and referral decision drivers for acceptable infusion sites (Data S1). Items were primarily 5‐point Likert scales (pain points: 1 = Not a problem to 5 = Severe; decision drivers: 1 = Not important to 5 = Extremely important), plus two open‐text questions. Pain points were rated in the context of respondents' real‐world experience arranging and maintaining timely infusions for pediatric patients with IBD across sites of care; respondents were not asked to attribute each barrier to a specific infusion setting.

The survey was distributed via a national pediatric GI listserv (membership >3500) from February 13–27, 2026. The invitation email was sent on February 13, 2026, and the survey remained open for 14 days. No reminder emails were sent. Participation was voluntary; no incentives were provided. Respondents were instructed that the survey was intended for United States‐based pediatric clinicians and that responses should reflect current practice. Reporting follows the Checklist for Reporting Results of Internet E‐Surveys (CHERRIES).^6^ Because listserv distribution does not provide a verifiable denominator of eligible recipients or survey exposure, response rates were not calculated. Consistent with the American Association for Public Opinion Research (AAPOR) standard definitions, response rate calculations require a defined sample frame and an approach to handling unknown eligibility; these were not available for listserv distribution.^7^

### 2.3 Use of artificial intelligence (AI) authoring tools

A generative AI tool (ChatGPT, OpenAI) was used to assist with language editing. All content was reviewed, edited, and verified for accuracy by the author. The tool was not used to generate study data or determine study conclusions, and no participant‐identifiable information was entered into the tool.

### 2.4 Statistical methods

We summarized categorical variables as counts and percentages. For Likert items, we calculated Top‐2 box proportions (ratings 4–5) and top box proportions (rating 5) with available‐case denominators; referral decision‐driver ratings are displayed in Data S2. Missing responses were not imputed. Open‐text responses were reviewed and coded inductively, then grouped into pragmatic themes; exemplar responses are provided in Data S3. Analyses were conducted in Python (Python Software Foundation).

## 3 RESULTS

A total of 100 responses were received (Table 1). Most respondents were physicians (92%), and 68% reported practicing in a children's hospital setting (multi‐select). When participants were asked to select the most common sites for receipt of infusions among their patients (multi‐select), hospital‐based pediatric infusion centers were selected by 76% of respondents, followed by independent/outpatient infusion centers (30%) and home infusion (20%). Fifty‐nine respondents (59%) provided an estimate of the proportion of their patients infused in non‐hospital settings; among these, 20.3% reported that more than half of their infused patients receive infusions in non‐hospital settings.

**Table 1:** Respondent characteristics and reported infusion site‐of‐care patterns.

Characteristic	Category	*n* (%)
Role	Physician	92 (92.0)
	Trainee	6 (6.0)
	APP	2 (2.0)
Practice setting (multi‐select)	Children's hospital	68 (68.0)
	Academic medical center (non‐children's hospital)	22 (22.0)
	Private practice	7 (7.0)
	Multispecialty group	5 (5.0)
	Other	1 (1.0)
Pediatric IBD panel size (<18 years)	300+	41 (41.0)
	150–299	24 (24.0)
	50–149	22 (22.0)
	<50	13 (13.0)
Most common infusion sites (multi‐select)	Office‐based infusion suite	10 (10.0)
	Hospital‐based pediatric infusion center	76 (76.0)
	Independent/outpatient infusion center	30 (30.0)
	Home infusion	20 (20.0)
	Adult hospital infusion center	6 (6.0)
	Other (free text)	3 (3.0)
	Other includes free‐text responses not matching prespecified categories (*n* = 3).	
Estimated proportion infused in non‐hospital settings	1%–10%	18 (30.5)
	11%–25%	17 (28.8)
	>50%	12 (20.3)
	26%–50%	9 (15.3)
	0%	3 (5.1)
	Item‐level *n* = 59; percentages use this denominator	

*Note*: Practice setting and infusion sites are multi‐select; percentages use *N* = 100 as the denominator unless otherwise indicated.

Abbreviations: APP, advanced practice provider; IBD, inflammatory bowel disease.

**Figure 1:**
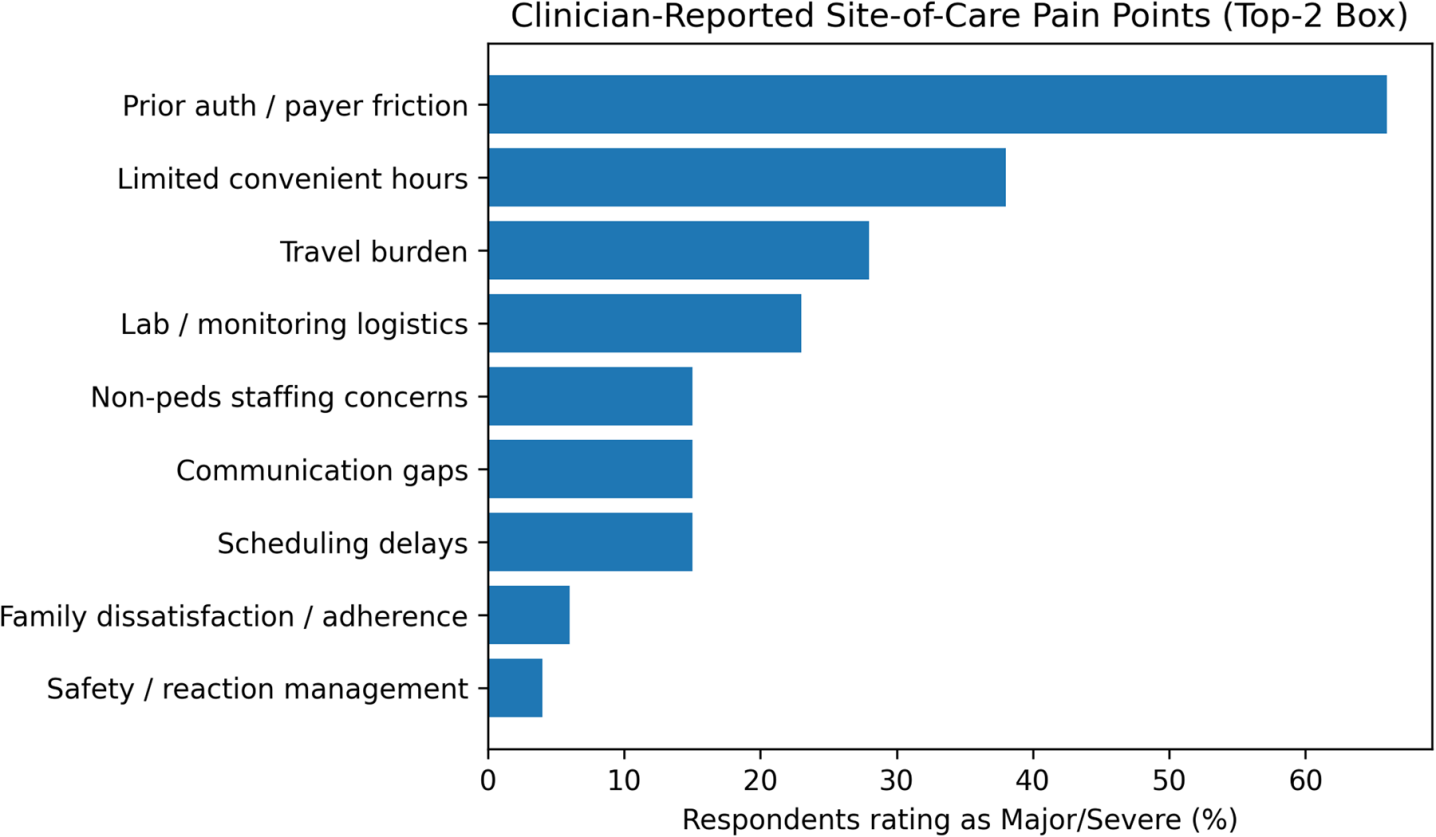
Reported access barriers (pain points) related to arranging and maintaining timely infusions among pediatric gastroenterology clinicians (Top‐2 box: ratings 4–5). Top‐2 box, proportion rating an item as major/severe (4–5 on a 5‐point Likert scale). Lab, laboratory; non‐peds, non‐pediatric; prior author, prior authorization.

Across assessed pain points related to arranging and maintaining timely infusions, prior authorization/payer friction was most frequently rated as a major/severe problem (66% Top‐2 box), followed by limited appointment availability (38%) and travel burden (28%) (Figure 1). In contrast, lack of confidence in safety/reaction management capability was infrequently rated as a major/severe pain point (4%).

Decision drivers for an acceptable infusion site were consistently high. The highest‐rated drivers were ability to handle infusion reactions/emergency protocols (88% very/extremely important; 70% extremely important), reliable communication back to the GI team (88%; 61% extremely important), patient experience/satisfaction (88%; 63% extremely important), and proven pediatric infusion expertise (87% among respondents with available data) (Data S2).

In open‐text responses, payer restrictions, prior authorization processes, and insurer‐directed site‐of‐care mandates were the most commonly cited access barriers. Respondents emphasized pediatric‐trained staff, rapid scheduling, and bidirectional communication as key elements of an ideal pediatric infusion experience (Data S3).

## 4 DISCUSSION

In this national convenience sample of pediatric GI clinicians, payer‐related administrative burden emerged as the dominant barrier to timely and coordinated pediatric infusion care, while safety capability and care integration (reaction management, pediatric expertise, and bidirectional communication) were consistently rated as highest‐priority attributes of acceptable infusion sites.

These findings align with pediatric IBD guidance emphasizing structured safeguards for non‐hospital biologic infusions, including pediatric‐specific training, standardized reaction protocols/emergency readiness, appropriate handling and storage of patient‐specific medications, and reliable communication pathways back to the treating team.^5^ Notably, the highest‐rated decision drivers in this survey map directly to key components of this guidance (pediatric‐trained staff, reaction management capability, and bidirectional communication), and they reinforce the importance of patient experience as a distinct domain when evaluating alternate sites of care. Prior pediatric IBD work has described quality improvement approaches to external infliximab infusions and standardized home infusion pathways, underscoring the importance of protocolization and communication when care moves outside hospital settings.^8^, ^9^ Patient expectations and satisfaction with biologic therapy have also been shown to depend on coordination, information, and service experience, supporting inclusion of patient experience as a core decision driver.^10^

This study has limitations. Recruitment through a specialty listserv yields an unknown eligible denominator and is subject to self‐selection bias; therefore, results may not be nationally representative. The survey was open for a limited period (14 days), which may preferentially capture respondents with higher salience or burden related to payer mandates. Eligibility (United States‐based practice) was requested but not independently verified. Responses were self‐reported and may reflect local payer environments and institutional processes. Item‐level nonresponse occurred for some questions (e.g., estimated proportion of non‐hospital infusions), and we used available‐case analysis without imputation. Finally, pain point items were not site‐specific; respondents were not asked to attribute barriers to particular infusion settings.

Future work should evaluate how payer‐directed site‐of‐care shifts affect measurable clinical outcomes (infusion delays, missed doses, acute care utilization) and patient‐ and caregiver‐reported experience, and should test standardized communication and escalation workflows between non‐hospital infusion sites and pediatric GI teams. A complementary caregiver/patient survey and prospective multicenter evaluations of pediatric‐specific infusion models could help define pragmatic quality metrics for contracting and network design.

## 5 CONCLUSION

In conclusion, pediatric GI clinicians report that payer‐related administrative barriers substantially influence pediatric infusion site‐of‐care decisions. When site‐of‐care shifts are unavoidable, payer and provider stakeholders should prioritize pediatric‐specific expertise, emergency readiness, and integrated bidirectional communication to reduce fragmentation, support adherence, and protect quality of care.

## CONFLICT OF INTEREST STATEMENT

The authors declare no conflict of interest.

## Supporting information

SDC 3 additional results.

SDC1_Survey Instrument.

SDC2 Referral Decisino Driver.
