# Supplementary material for: Clinician perspectives on infusion site‐of‐care shifts in pediatric inflammatory bowel disease: An anonymous national web‐based survey
Source: JPGN Rep. 2026 Sep 1:10.1002/jpr3.70238. Online ahead of print. doi: 10.1002/jpr3.70238 (PMC13530913; doi:10.1002/jpr3.70238)
Supplement: Supplementary file 1 [file JPR3-9999-0-s001.docx]

**Supplemental Digital Content 2. Referral decision drivers for an acceptable infusion site, shown as the proportion of respondents rating each driver as very/extremely important (top-2 box; Likert 4–5).**
