# Supplementary material for: Clinician perspectives on infusion site‐of‐care shifts in pediatric inflammatory bowel disease: An anonymous national web‐based survey
Source: JPGN Rep. 2026 Sep 1:10.1002/jpr3.70238. Online ahead of print. doi: 10.1002/jpr3.70238 (PMC13530913; doi:10.1002/jpr3.70238)
Supplement: Supplementary file 2 [file JPR3-9999-0-s002.docx]

**Supplemental Digital Content (SDC) 1. Survey Instrument**

**Pediatric GI Providers: “Referral Drivers & Service Needs”**

**INTRO**

We’re gathering input from pediatric GI clinicians to understand infusion access challenges and what an ideal pediatric-focused infusion option should provide. This survey is anonymous and takes ~6 minutes.

**PRACTICE CONTEXT**

1) Your role:

- Physician

- APP (NP/PA)

- RN/Infusion coordinator

- Pharmacist

- Other: ________

2) Practice setting:

- Children’s hospital employed faculty

- Private practice

- Academic medical center (non-children’s)

- Multispecialty group

- Other: ________

3) Approximate number of pediatric IBD patients you manage:

- <50

- 50–149

- 150–299

- 300+

4) For infused therapies in your practice, where do patients MOST commonly receive infusions today? (Select up to 2)

- Hospital-based pediatric infusion center

- Adult hospital infusion center

- Independent/outpatient infusion center

- Office-based infusion suite

- Home infusion

- Mixed / varies widely

**CURRENT PAIN POINTS**

(Scale: 1=Not a problem, 2=Minor, 3=Moderate, 4=Major, 5=Severe)

5) Scheduling delays (time-to-infusion)

6) Limited appointment availability (after school/weekends)

7) Patient travel burden

8) Prior authorization / payer friction

9) Lab coordination / therapeutic monitoring logistics

10) Communication gaps between infusion site and GI team

11) Concerns about pediatric-specific staffing/experience in non-peds settings

12) Site-of-care safety protocols / reaction management confidence

13) Family dissatisfaction affecting adherence / missed doses

**REFERRAL DECISION DRIVERS (importance weights)**

(Scale: 1=Not important … 5=Extremely important)

14) Proven pediatric infusion expertise

15) Fast time-to-appointment

16) Reliable communication back to GI team (results/issues)

17) Ability to handle infusion reactions / emergency protocols

18) Geographic convenience for families

19) Payer acceptance / contracting breadth

20) Predictable patient out-of-pocket costs / transparency

21) Consistent processes (orders, labs, documentation)

22) Patient experience / satisfaction

**CONCEPT TEST**

(Show this text block before Q23)

Consider a pediatric-focused outpatient infusion center with pediatric-trained nurses, child-friendly environment, standardized safety protocols, efficient lab/order coordination, and rapid communication to the GI team. Goals include reducing scheduling delays and family burden.

23) If this option were available and contracted with major payers, how likely are you to refer appropriate patients?

- Very unlikely

- Unlikely

- Not sure

- Likely

- Very likely

24) What percentage of your infused patients might be appropriate to shift to a pediatric-focused outpatient infusion center (assuming payer coverage and comparable safety)?

- 0%

- 1–10%

- 11–25%

- 26–50%

- >50%

- Not sure

**ADOPTION BARRIERS (Select all that apply)**

25) What would most limit your referrals?

- Payer/site-of-care restrictions

- Concerns about emergency preparedness

- Concerns about pediatric staffing/training

- Poor communication back to GI team

- Unclear lab/monitoring workflow

- Patient preference to stay where they are

- Administrative friction (orders, documentation)

- Other: ________

**WHAT “PROOF” YOU’D NEED**

26) Which metrics would most convince you the model is working? (Select up to 5)

- Shorter time-to-infusion (days)

- Fewer missed/delayed infusions

- Patient satisfaction scores

- Improved adherence/persistence on therapy

- Lower ED visits related to infusion issues

- Faster lab result turnaround

- Lower family travel time/burden

- Lower total cost of care

- Other: ________

**OPEN TEXT (quotable)**

27) In one sentence: what is the biggest infusion access problem for your patients today?

[free text]

28) If you could design the “ideal” pediatric infusion experience, what must it include?

[free text]
