# Supplementary material for: Clinician perspectives on infusion site‐of‐care shifts in pediatric inflammatory bowel disease: An anonymous national web‐based survey
Source: JPGN Rep. 2026 Sep 1:10.1002/jpr3.70238. Online ahead of print. doi: 10.1002/jpr3.70238 (PMC13530913; doi:10.1002/jpr3.70238)
Supplement: Supplementary file 3 [file JPR3-9999-0-s003.docx]

**Supplemental Digital Content 3. Additional Results**

| Question / Theme | Representative excerpts |
| --- | --- |
| Biggest access problem Theme: Insurance / payer mandates | • Insurance is usually a bigger challenge than the disease that we are trying to treat • Insurance deciding where to send them • Limited locations and insurance limitations for private home infusion partners. |
| Ideal pediatric infusion experience Themes: Pediatric expertise, convenience, emergency readiness | • Pediatric trained nurses, friendly environment that caters to pediatric patients, and is conducive to a family centered approach • Close to home with pediatric trained staff with convenient access. • Pediatric only infusion suites, ability to respond to emergencies |
